# Paranoia and the social representation of others: a large-scale game theory approach

**DOI:** 10.1038/s41598-017-04805-3

**Published:** 2017-07-03

**Authors:** Nichola J. Raihani, Vaughan Bell

**Affiliations:** 1 Department of Experimental Psychology, University College London, London, United Kingdom; 2 Division of Psychiatry, University College London, London, United Kingdom; 3 South London and Maudsley NHS Foundation Trust, London, United Kingdom

## Abstract

Current definitions of paranoia include two key components: unfounded ideas of harm and the idea that the harm is intended by others. However, attributions of harmful intent have been poorly studied and mainly using artificial scenarios rather than participation in genuine social interactions where genuine resources are at stake. Using a large non-clinical population (N = 3229) recruited online, we asked people to complete a measure of paranoid ideation before playing a modified Dictator Game, where the ‘dictator’ can allocate money to the partner (the ‘receiver’). Participants were allocated to the role of receiver or of an uninvolved observer; and evaluated to what extent they believed dictator decisions were motivated by (i) self-interest or (ii) harmful intent. All participants attributed more harmful intent to unfair as opposed to fair dictators. Paranoia had a positive effect on harmful intent attribution, for both fair and unfair dictators. Paranoia did not interact with attributions of self-interest. Importantly, highly paranoid participants attributed equally strong harmful intent to the dictator in the observer role as in the receiver role. This challenges the assumption that paranoia is mainly due to an exaggerated sense of personalised threat and suggests instead that paranoia involves a negative social representations of others.

## Introduction

Paranoia involves two key components: a person having unfounded ideas that harm will occur to them, and the idea that the harm is intended by others^1^. Current accounts of the formation and maintenance of paranoia include a cognitive style involving worry, negative thoughts about the self, interpersonal sensitivity, anomalous experiences, insomnia and reasoning biases as key components^2^. The threat anticipation model suggests that these converge and lead to an over-interpretation of potential harm to the self – a state that self-maintains in people with high levels of paranoia primarily due to anxiety-driven avoidance of disconfirmatory evidence^3, 4^.

Differences in threat perception have been well-established and studies have consistently found threat-related material engages attention to a far greater degree in clinically paranoid people (reviewed in ref. 5). It is unlikely that this is explained by differences in general ‘theory of mind’ ability, despite the obvious misattributions of mental state. Although past studies include both positive and negative findings^6^, a meta-analysis of theory of mind in schizophrenia reported no overall association with paranoia^7^. However, numerous studies have investigated attributional biases in the interpretation of events, and have shown a general tendency for attributing responsibility to other people rather than situations for negative events^8^.

Notably, attributions of whether harm is intended, rather than where responsibility lies for negative events, have been far less studied despite this being a key aspect of paranoia. Studies using the Ambiguous Intentions Hostility Questionnaire^9^ have reported that people with high levels of non-clinical and clinical paranoia are more likely to rate vignettes describing ambiguous scenarios as involving hostile intent^9–12^. More recently, Freeman *et al*.^13^ used a paradigm from Ames and Fiske^14^ to test differing attributions from vignettes describing intentional and unintentional harm with identical consequences. Counter to the researchers’ predictions, they found that people with high levels of paranoia were less likely to make attributions of intent and blame in the vignette describing intentional harm than people with low levels of paranoia but more likely to ascribe blame and intent in the vignette describing non-intentional harm. However, they suggested that these third-person vignettes may not have sufficiently engaged self-relevant concerns, and that if participants were directly involved in an interaction, highly paranoid people might show a more typically paranoid attributional style where intent to harm would be more strongly attributed.

This speaks to a significant limitation of these studies, in that they typically involve rating hypothetical scenarios describing interactions in the third-person, rather than genuine social interactions where the attribution of intent is directly relevant to resolving the interaction as it occurs^15^. To address some of these issues, Ellett *et al*.^16^ used an interactive ‘Prisoner’s Dilemma’ game^17^ to test whether people with high levels of paranoid ideation were more likely to use competitive rather than cooperative strategies when playing against others. They reported that high levels of paranoid ideation predicted competitive strategy use and – importantly – that competitiveness was better predicted by distrust than by self-interest among paranoid participants.

Paradigms taken from game theory are now widely used in social cognition research and are starting to be used more frequently in psychopathology research^18^. Although several such studies have investigated strategic decision-making in people diagnosed with schizophrenia (e.g., refs 19–21) or with varying levels of schizotypy^22, 23^, as far as we know, only Ellett *et al*.^16^ have specifically focused on paranoia. Moreover, until now, no studies have combined measures of social decision-making with measures of intent attribution, both of which are needed to tackle the two key components of paranoia.

We aimed to extend this research by using a social interaction with real monetary stakes and a measure of the perceived intentions of the partner, to test the effect of differing levels of paranoid ideation on intent attributions in a large general population sample. Online participants were paired to play the ‘Dictator Game’^24^ for real money. This is a widely used economic task which measures other-regarding preferences in the absence of strategic incentives to give. In this task, one player (the ‘dictator’) is given a sum of money which they can choose to share with their partner (the ‘receiver’). The receiver has no control and must accept any amount that the dictator offers. Theoretical predictions based on short-term, payoff-maximising preferences state that dictators will keep all the money – but this prediction is frequently refuted. In fact, the mean donation in this task is 28% of the endowment^25^, indicating that people harbour economically ‘irrational’, yet prosocial preferences.

Unlike several other game theory paradigms, participation is non-reciprocal, meaning that cooperative or uncooperative behaviour cannot be interpreted as a response to, or an attempt to influence, the partner’s behaviour. Moreover, the payoffs in a Dictator Game are determined entirely by the dictator, which rules out the possibility that dictator decisions are based on the anticipated responses of their partners, which would introduce other within-game strategic motivations.

The Dictator Game is particularly suited to paranoia research as the motives underpinning decision-making in this task are ambiguous with respect to harmful intent. A dictator who keeps (or takes) the entire endowment might do so because they wish to maximise their own earnings in the task. In other words, they might be motivated by self-interest. Indeed, this is the implicit assumption underpinning the canonical model of human behaviour in such games^26^. However, it is also possible (if less plausible) that selfish dictator decisions are motivated by a desire to harm the partner, by denying them of earnings in the task. Ambiguity in task performance has been shown to increase paranoid ideation^27^ and the difficulty involved in evaluating intentions in ambiguous situations results in stronger attributional hostility among persons with higher levels of paranoia^9, 10^. Accordingly, we were interested in whether people high in paranoia would attribute higher degrees of harmful intent to the dictator when compared to people lower in paranoid ideation. Importantly, we also included an observational third-person paradigm where participants were asked to evaluate harmful-intent when they were solely an observer to a Dictator Game interaction, to test whether being uninvolved would reduce attributions of harmful intent for people with high levels of paranoia ideation, as predicted by Freeman *et al*.^13^.

## Methods

This project was approved by the University College London ethics board under project number 3720/001. The study was carried out in accordance with the relevant guidelines. Prior to taking part in the study, participants were informed that their participation was voluntary and were required to tick a box that consented to the authors using their anonymous data for research purposes. Participants were also given the opportunity to sign up to an email list to find out more about the study’s objectives after they had participated. All data were collected in December 2016 using Amazon Mechanical Turk (hereafter MTurk; http://www.mturk.com), an online crowdsourcing platform. This allowed us to recruit a more demographically diverse sample of participants than would have been possible had we relied on undergraduate participant pools^28^ without sacrificing data quality (since it has been demonstrated several times that data collected via MTurk produces similar or more accurate results to those obtained under traditional laboratory settings^29–31^).

We recruited 3,229 participants (1,695 females, 1,530 males, 4 did not specify) to the study. The mean age of the participants was 36 ± 0.2 years (range: 18–80 years). Participants first completed the Green *et al*. Paranoid Thoughts Scale (hereafter GPTS; ref. 32) a reliable and valid scale for measuring paranoia across the clinical spectrum. This 32-item scale assesses ideas of social reference and persecution and, importantly, has been shown to be a reliable and valid measure of the continuum of paranoid thoughts in both clinical and non-clinical populations. Participants were asked to indicate the extent of feelings described in 32 statements using a Likert Scale of 1 to 5, where 1 = Not at All and 5 = Totally. Scores can range from 32–160, with higher scores indicating a greater degree of paranoia. The GPTS was chosen as a suitable measure as it includes both core aspects of the definition of paranoia^1^: social concerns about others and perception of intended harm. The total paranoia score was obtained for each participant by summing the response scores to all questions, comprising both the social reference and the persecution scales. Hereafter, this variable is referred to as ‘paranoia’.

After completing the survey, we allowed a minimum interval of 5 days to elapse before inviting participants to take part in a Dictator Game, either in the role of receiver (n = 1,274) or in the role of an uninvolved observer (n = 1,123) (see below). The internal and external validity of the Dictator Game as a measure of prosocial preference has been demonstrated previously, both in lab settings and in the MTurk online laboratory (see ref. 33 and references therein). The discrepancy between the number of participants who took the survey and the total number of participants who played a Dictator Game arose due to attrition (participants not responding to the call-back) and failed comprehension question attempts (which resulted in exclusion from the task). As participants who failed the comprehension questions were not included in the study, no data was recorded from them, and so it is not possible to distinguish what proportion of attrition was due to non-response and what proportion due to non-comprehension.

We made two important modifications to our Dictator Game task. The endowment at stake was $0.50, but rather than allowing dictators to share any amount with the receiver, we limited them to making a fair decision ($0.25–$0.25 split) or an unfair decision ($0.50 for dictator; $0.00 for receiver) (c.f. ref. 34). In addition, we randomly allocated dictators to a ‘give’ or a ‘take’ frame (following^35^). In the give frame, dictators were given the $0.50 endowment and could send half or none to the receiver. In the take frame, the receiver was given the endowment and the dictator could take half or all of it from the receiver. Thus, although the payoff consequences of fair and unfair decision-making were the same in the give and take frames, the manner in which these outcomes were achieved was different, and might be interpreted as such by participants. In our study, dictators were referred to as ‘Player 1’ and receivers as ‘Player 2’. The terms ‘fair’ and ‘unfair’ were not used in the instructions seen by players; instead, dictator allocations were simply described in terms of their actions and consequences (e.g. “Player 1 kept $0.25 and sent $0.25 to Player 2”). Sample game instructions are available as supplementary materials.

In one Dictator Game, participants first played in the role of receiver – this is hereafter referred to as the ‘second-party Dictator Game’. In the ‘third-party Dictator Game’, participants initially played in the role of an observer, who witnessed the interaction between a dictator and receiver. Thus, in the second-party Dictator Game, the participant was directly involved, whereas in the third-party Dictator Game, they participated as an uninvolved bystander. After finding out the condition (giving/taking) and the fairness of the dictator’s decision (fair/unfair), receivers and observers, respectively, were asked to complete two ratings (using slider bars initialised at 50) on a scale of 1 to 100 to what extent they believed the dictator’s decision was motivated (i) by a desire to earn more, and (ii) by the dictator’s desire to reduce the participant’s bonus (or the bonus of the receiver, in the third-party Dictator Game). The first rating therefore corresponds to inference that the dictator was motivated by self-interest, while the second captures the extent to which dictators were inferred to be motivated by desire to harm the partner. Thus, receivers made inferences about the dictator when they were the partner of that individual, whereas observers made inferences about the dictator when they were an uninvolved third-party.

After making their inferences, all players subsequently were allocated to the dictator role and were asked to decide whether to make a fair/unfair allocation to their partner in a giving/taking condition. These dictator decisions were not used for analysis but were collected so that we could truthfully inform participants in the first phase of the game that all other players were real and that the dictator decisions they witnessed had been made by other participants. In the third-party Dictator Game, the participants were also informed (at the end of the task) that they were also the receiver in a game with a different dictator – meaning that the decisions they made as dictators had meaningful payoff consequences for other players. Ex-post matching (c.f. refs 29, 34 and 36) was used to assign partners.

The motives underpinning decision-making in the Dictator Game task are ambiguous with respect to harmful intent versus self-interest. Given this ambiguity, we had no a priori expectations about whether dictators would be, on average, rated as more motivated by self-interest or a desire to cause harm, respectively. However, we did expect that i) increased paranoia would be associated with an increased tendency to attribute harmful intentions to dictators and, based on the predictions of Freeman *et al*.^13^ we expected that ii) this effect would be most pronounced when participants were cast in the role of receiver than as an (uninvolved) observer. We also predicted that paranoia would interact with the fairness of the dictator decision (paranoid participants would attribute more harmful intentions to unfair than to fair dictators) and with the frame (paranoid participants would attribute more harmful intentions in the take than in the give frame). Finally, we anticipated a three-way interaction between paranoia, frame and fairness, expecting the strongest harm inference to come from paranoid individuals, who were faced with an unfair dictator in the take frame.

We had different predictions with respect to inferences that dictators were self-interested. We did not expect that paranoia would influence the self-interest inference. Rather, we expected that participants would simply attribute stronger self-interested intentions to unfair than to fair dictators. We did not expect the frame (give/take) to influence the self-interest inference; nor did we expect the interaction between frame and paranoia to be meaningful for this inference.

### Statistical approach

We used an information-theoretic approach with multimodel averaging^37^. An information-theoretic approach has several advantages over traditional frequentist approaches (outlined in ref. 38). Rather than focusing on arbitrary p values as arbiters of significance, under this philosophy, a predefined set of candidate models (containing different numbers and or combinations of explanatory terms) are simultaneously compared to one another and the relative support for each model is considered, given the fit to the data. The support for each model is determined by comparing the Akaike Information Criterion corrected for small sample sizes (AICc^39^), with lower AICc values indicating greater support for that model. If several models have a similar level of support, then a model-averaging approach allows us to take the resulting uncertainty over which is the “best” model into consideration when calculating effect sizes, providing more conservative estimates. An information-theoretic approach also circumvents the often overlooked problem of multiple hypothesis testing that occurs when performing stepwise backwards regression to derive the best (minimal) model from full models (see ref. 40).

We conducted two broad analyses with our dataset: one to determine when harmful intentions were attributed to dictators, and another to determine when dictators were viewed as being self-interested. For each analysis, we first specified a global model, containing all fixed effects and interactions that were deemed to be of significance (see below). All continuous input parameters were centred and standardized dividing by two standard deviations (following^41^). Binary input variables were centred by subtracting their mean. This means that model parameter estimates can be interpreted on the same scale. The global model was refined using the *dredge* function in MuMin^42^ which compares all possible models and yields a top model set, which contains all the models that are within 2 AICc units of the ‘best’ model (that with the lowest AICc value). To obtain parameter estimates, we averaged across this top model set – this approach takes into account the uncertainty over the true parameter estimate when many models have similar levels of support. All estimates reported here are full model averages, which provide conservative estimates for terms that are not included in all of the top models.

Since attributions made about dictators were extremely skewed, we converted each response variable (cause harm inference and self-interest inference) into a 10-level, ordered categorical variable. Each of these dummy variables was set as the response term in an ordinal logistic regression model (using the *clm* function in the ordinal package^43^) investigating attributions of harmful intent and self-interest for dictators, respectively. In each model, we specified the following input variables: age, fairness (fair/unfair), frame (give/take), gender, paranoia (a continuous input variable) and role (receiver/observer). We also included two-way interactions between the following terms: “fairness × frame”, “fairness × paranoia”, “fairness × role”, “frame × paranoia”, “role × paranoia”, and the following three-way interactions: “fairness × role × paranoia” and “fairness × frame × paranoia”. For each analysis, we display the top model set produced and full-model averaged estimates and confidence intervals (which yield conservative estimates for terms that are not included in all models in the top model set).

### Data Availability

All data and R code to reproduce analyses are available at^44^ https://figshare.com/s/f18a603bbed3a40e6124.

## Results

The average score on the social reference scale was 27.3 ± 0.24 (range: 16–80); and on the persecution scale was 23.4 ± 0.24 (range: 16–80). The resulting average score for the combined paranoia scale was 50.7 ± 0.47 (range: 32–160). Average scores on the social reference subscale were significantly higher than for those on the persecution subscale (paired t-test: t = 26.3, df = 2394, p < 0.001). The mean score for attributing self-interested motives to dictators was 72.5 ± 0.68, and for attributing harmful intent was 20.5 ± 0.58 – inferences regarding self-interest were significantly stronger than those regarding harmful intent (Wilcoxon signed rank test, V = 2248200, p < 0.001).

**Figure 1:**
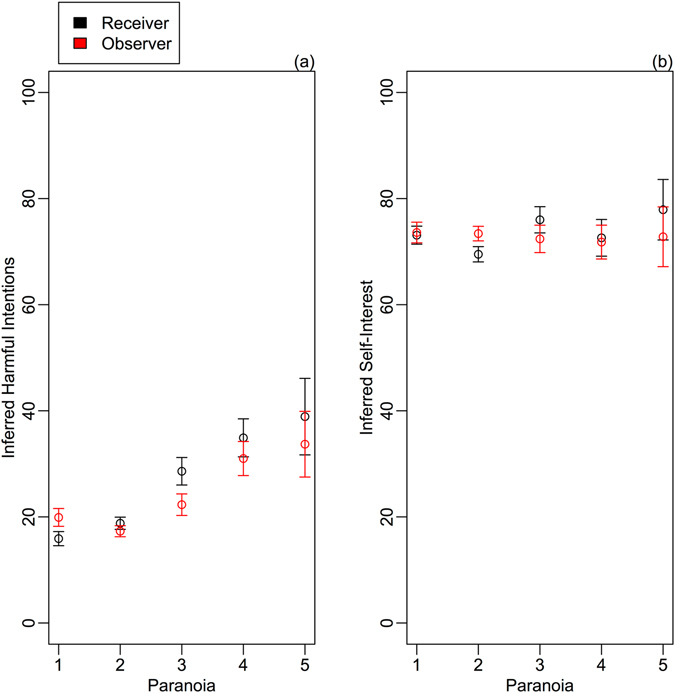
Attributions of (**a**) harmful intent and (**b**) self-interest for dictators as a function of paranoia ideation. Black circles represent attributions made in the receiver role; red circles represent attributions made in the observer role. Means and standard errors are generated from raw data. For visualisation, paranoia here is shown as a 5-level categorical dummy variable (where *1* ≤ *35*; *36* < *2* ≤ *60*; *61* < *3* ≤ *85*; *86* < *4* ≤ *110*; *111* < *5* ≤ *160*). Please note: In the statistical analyses, paranoia is treated as a continuous term (though analyses are robust when paranoia is included as a categorical variable).

**Figure 2:**
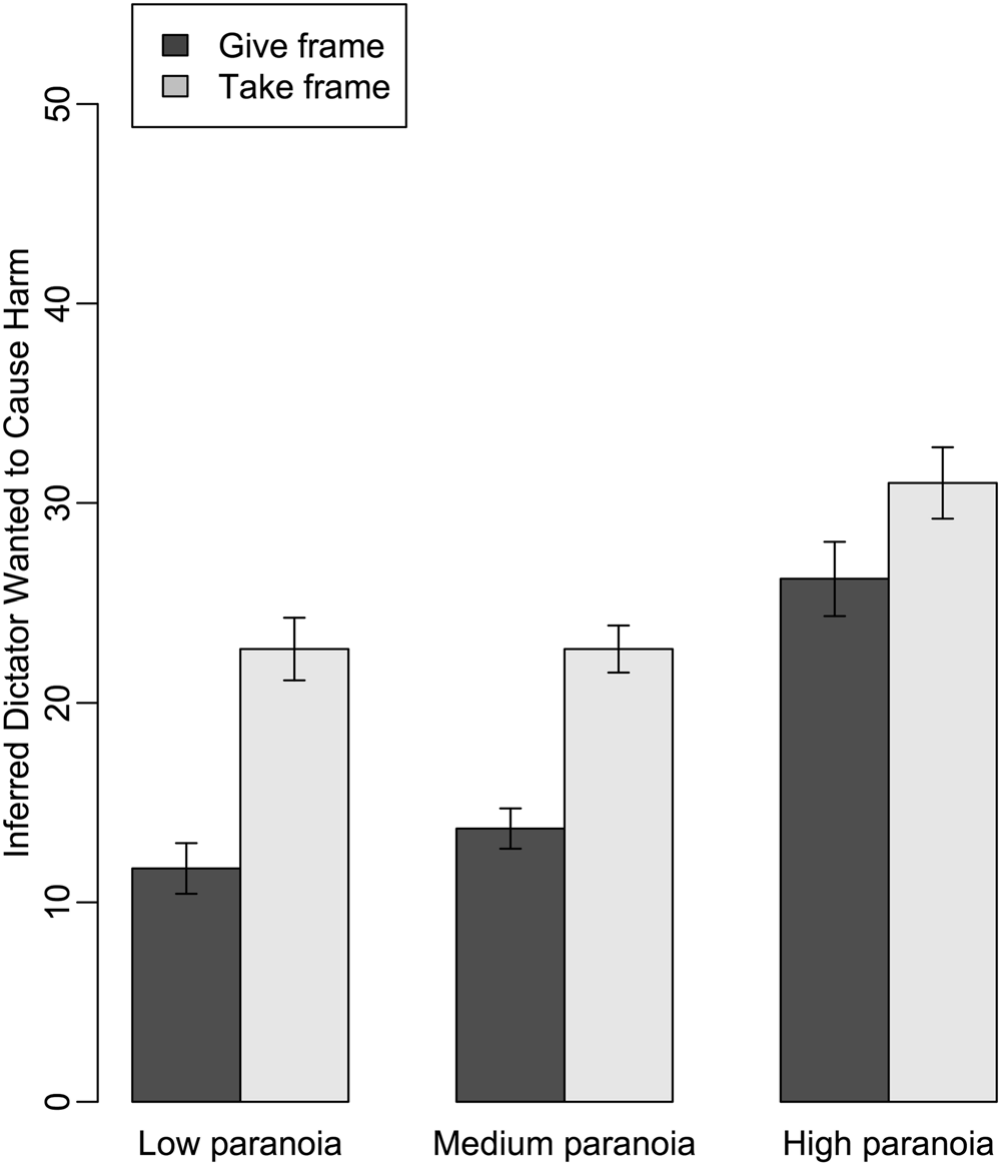
Attributions of harmful intent to dictators as a function of the game frame (give/take) and the paranoia ideation of the participant. Please note: For visualisation, paranoia is shown as a 3-level categorical variable (*low* ≤ *35*; *36* < *medium* ≤ *59*; *60* < *high* ≤ *160*). Means and standard errors are generated from raw data.

High paranoid ideation was associated with greater attributions of harmful intent to dictators (estimate: 0.83; CI: 0.66, 1.00; Table S1; Table 1; Fig. 1a). Participants also attributed more harmful intent to unfair than to fair dictators (estimate: 0.56; CI: 0.39, 0.74); and when playing against dictators in the take frame than in the give frame (estimate: 0.77; CI: 0.59, 0.95), regardless of whether the dictator was fair or unfair (Fig. 2). Interestingly, participants that were high in paranoid ideation made stronger inferences about dictators’ harmful intent in both the give and take frames, whereas those who were lower in paranoid ideation made weaker inferences overall and showed a greater discrimination between the give and take frame (Fig. 2). Women also inferred greater harmful intentions than men (estimate: −0.33; CI: −0.50, −0.16); and attributions of harmful intent were stronger among younger players than older (estimate: 0.22; CI: 0.05, 0.40). Counter to our predictions, we found no effect of role or of a role x paranoia interaction on attributions of harmful intent (Fig. 1a). This indicates that, regardless of whether they play as an involved recipient or an uninvolved observer, participants make the same inferences about dictators’ harmful intentions and – moreover – that the tendency for those that are high in paranoid ideation inference to attribute harmful intent is extended to scenarios where the subject is not the target of the purportedly harmful action.

**Table 1:** Factors affecting attribution of harmful intent.

Parameter	Estimate	Unconditional SE	Confidence Interval	Relative Importance
*Intercept 1|2*	*0*.*73*	*0*.*05*	(*0*.*63*, *0*.*82*)	
*Intercept 2|3*	*1*.*60*	*0*.*06*	(*1*.*49*, *1*.*71*)	
*Intercept 3|4*	*2*.*14*	*0*.*07*	(*2*.*01*, *2*.*27*)	
*Intercept 4|5*	*2*.*82*	*0*.*09*	(*2*.*66*, *2*.*99*)	
Age	0.22	0.09	(0.05, 0.40)	1.00
Fairness (fair)	0.56	0.09	(0.39, 0.74)	1.00
Frame (give)	0.77	0.09	(0.59, 0.95)	1.00
Gender (female)	−0.33	0.09	(−0.50, −0.16)	1.00
Paranoia	0.83	0.09	(0.66, 1.00)	1.00
Frame:Paranoia	−0.52	0.16	(−0.84, −0.20)	1.00
Fairness:Frame	−0.27	0.21	(−0.68, 0.13)	0.81
Role (observer)	−0.03	0.08	(−0.11, 0.18)	0.66
Paranoia:Role	0.18	0.20	(−0.21, 0.58)	0.57
Fairness:Paranoia	−0.03	0.10	(−0.23, 0.16)	0.22
Fairness:Role	0.02	0.09	(−0.15, 0.19)	0.13

Model averaged estimates, unconditional standard errors, confidence intervals and relative importance for the terms included in the top model set (Table S1). The response term for the model was a five-level, ordered categorical variable, indicating the extent to which participants attributed harmful intent to dictators. For categorical variables, reference levels are shown in parentheses. Input variables were scaled so estimates can be considered on the same scale.

**Figure 3:**
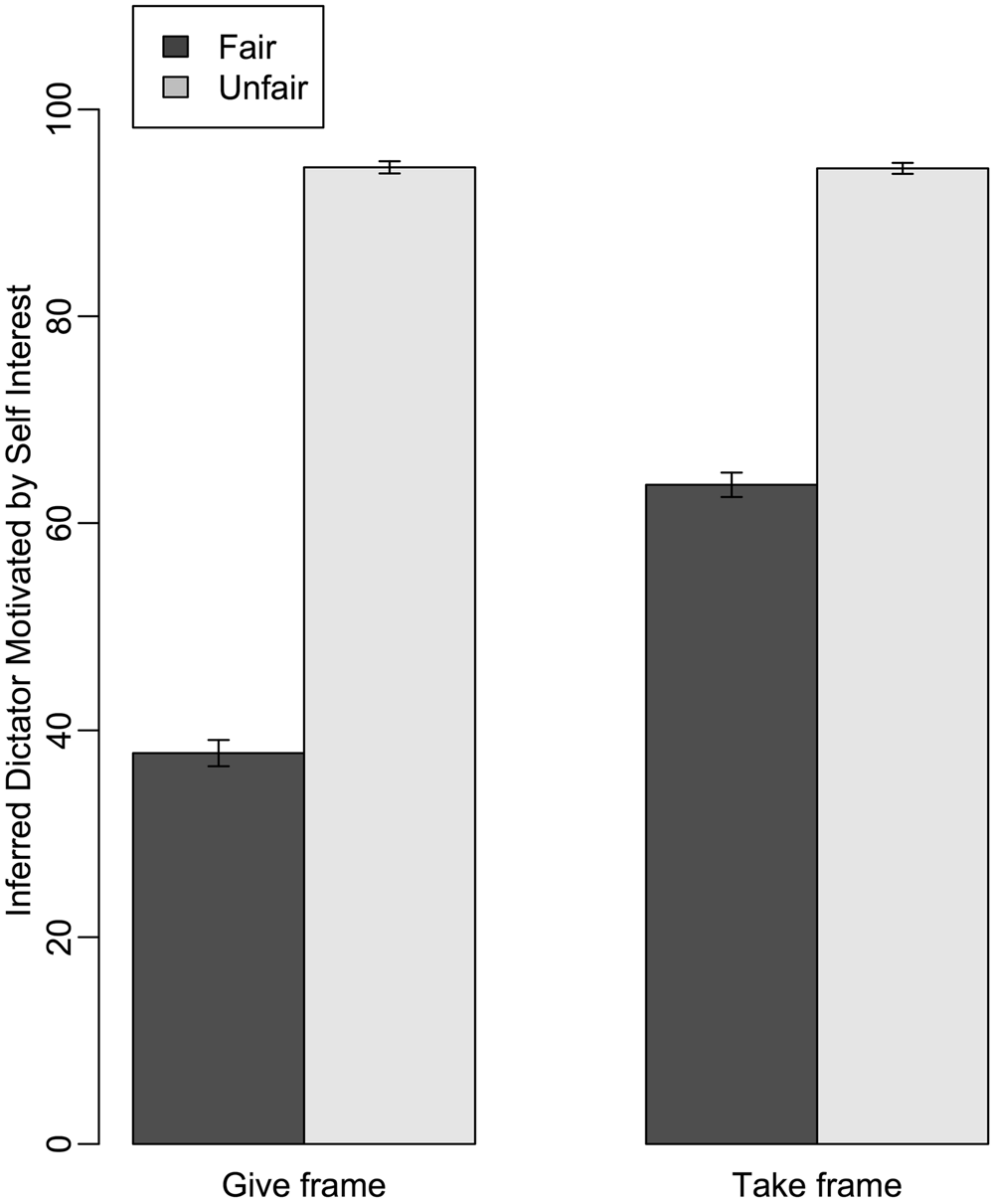
Attributions of self-interest for dictators as a function of the dictator’s decision and the game frame. Means and standard errors are generated from raw data.

By contrast, attribution of self-interest to dictators did not vary systematically with paranoia score (estimate: −0.13; CI: −0.32, 0.07; Table S2; Table 2; Fig. 1b), though our analyses did reveal an interaction between paranoia and dictator fairness on attributions of self-interest. A comparison of raw means (not controlling for other terms in the model) indicates that participants in the upper quartile of paranoia scores were more likely than those with lower paranoia scores to attribute self-interested intentions to fair dictators. As expected, participants were most likely to attribute self-interested motives to dictators when the dictators were unfair than when they were fair (estimate: 3.74; CI: 3.50, 3.98). There was also an interaction between fairness and frame, with dictators being viewed as increasingly self-interested in the take frame than the give frame, even when the dictators made a fair decision (Table S2; Table 2; Fig. 3).

**Table 2:** Factors affecting attribution of self-interest.

Parameter	Estimate	Unconditional SE	Confidence Interval	Relative Importance
*Intercept 1|2*	−*3*.*22*	*0*.*09*	(−*3*.*39*, −*3*.*05*)	
*Intercept 2|3*	−*2*.*31*	*0*.*08*	(−*2*.*45*, −*2*.*16*)	
*Intercept 3|4*	−*1*.*50*	*0*.*07*	(−*1*.*64*, −*1*.*40*)	
*Intercept 4|5*	−*0*.*39*	*0*.*06*	(−*0*.*51*, −*0*.*27*)	
Fairness (fair)	3.74	0.11	(3.50, 3.98)	1.00
Frame (give)	0.77	0.11	(0.55, 0.99)	1.00
Paranoia	−0.13	0.10	(−0.32, 0.07)	1.00
Fairness:Frame	−1.44	0.22	(−1.88, −1.00)	1.00
Fairness:Paranoia	−0.87	0.20	(−1.26, −0.49)	1.00
Role (observer)	−0.01	0.05	(−0.10, 0.08)	0.18
Frame:Paranoia	−0.02	0.09	(−0.19, 0.15)	0.17
Gender (female)	0.00	0.04	(−0.08, 0.07)	0.14
Age	0.00	0.04	(−0.07, 0.07)	0.14

Model averaged estimates, unconditional standard errors, confidence intervals and relative importance for the terms included in the top model set (Table S2). The response term for the model was a five-level, ordered categorical variable, indicating the extent to which participants attributed self-interest to dictators. Input variables were scaled so estimates can be considered on the same scale.

## Discussion

We investigated the effect of paranoid ideation on the attribution of harmful intent and self-interest using a novel application of the Dictator Game. Two versions were used: one where the participant was an active participant and one where the participant was an uninvolved observer. In agreement with our first main prediction, we found that paranoid ideation was positively associated with attribution of harmful intent, regardless of the dictator’s generosity in dividing the available money. Contrary to our second main prediction, however, people high in paranoia attributed equally high levels of harmful intent regardless of whether they were affected by the dictator’s harmful decision or were only observing the interaction, suggesting a view of others as generally intending harm regardless of the immediate threat to the self. Finally, regardless of paranoid ideation, all subjects made stronger attributions of self-interest for selfish dictators. Thus, paranoia does not seem to universally affect social evaluations but is instead specific to evaluations of harmful intent, as predicted.

Traditionally, much research in paranoia has focused on biases in the interpretation of events, particularly as they relate to threats to the self^3^. This is an implicitly individualistic approach where paranoia is thought to primarily reflect an ongoing sense of self-focused threat arising partly as information is ‘filtered’ through cognitive biases and partly as this cognitive style is maintained by worry, poor sleep, disturbed affect and unhelpful coping^2^.

Our findings challenge this approach and suggest an additional factor where paranoia involves, to a significant degree, biases in the representation of other social agents, independent of their immediate threat to the individual – in line with the social agent representation approach to psychosis^45^. This also echoes Chadwick’s^46^ distinction between symptomatic and schematic paranoia, where the former describes paranoid beliefs or thoughts that may wax and wane, and the latter a more profound alteration in social world-view.

Although models of paranoia have mentioned differences in the schematic representation of others^3, 47^, this is still an under-investigated area. The few studies that have tackled it, however, suggest it may be one of the strongest predictors of paranoia and related psychosis-spectrum symptoms. Fowler *et al*.^48^ reported that beliefs about others as negative (hostile, devious, harsh etc) as measured by the Brief Core Schema Scales were the strongest predictor of paranoia and above negative beliefs about the self, something also found in an earlier study using the Evaluative Beliefs Scale^49^. Negative-other schema were the single biggest predictor of paranoia (above other schematic beliefs, hallucinatory experience, and trauma symptoms) in a study of non-clinical participants^50^. In a clinical high risk of psychosis group, Addington and Tran^51^ reported that negative-other beliefs were the strongest predictor of unusual thought content, suspiciousness and total positive symptom score. Though suggestive, these previous studies relied on self-report measures of participants’ beliefs.

By contrast, here we directly elicited attributions in social exchanges for which genuine resources were at stake in a very large sample, one of the only studies to have taken this approach. The previous study using a game theoretic paradigm suggested that reduced cooperation in a Prisoner’s Dilemma Game could be attributed to reduced trust in a social partner^16^. Our data build on these findings by showing that paranoia does not just involve judgments about how the partner will treat self – but instead involves more general negative social representations of others. Indeed, this echoes previous findings by Paget and Ellett^52^ who examined the representation of the perceived persecutor in paranoia in relation to the self and others, finding that it was demarcated by perceptions of omnipotence and malevolence, the latter of which predicted delusional conviction. Our data therefore provide an important validation for the representation of others as an important factor in paranoia.

The game frame (give/take) also affected attribution of harmful intent and interacted with paranoia in the predicted manner. Subjects at the low and middle end of the paranoia spectrum attributed more harmful intent to dictators in the take frame than the give frame, whereas those who scored high for paranoid ideation made stronger harmful intent inferences overall while appearing to be less sensitive to the game frame – again suggesting that paranoia led to attribution of harm regardless of context.

We also found an unexpected interaction that didn’t involve paranoia between the frame (give/take) and the dictator’s decision (fair/unfair) on attributions of self-interest: specifically, participants made stronger attributions of self-interest in the take frame, even when dictators were fair. Framing effects have been investigated in Dictator Games several times before, with some studies finding that people behave more prosocially in a take frame^53^ and others finding no effect of the frame on generosity^34, 54, 55^. Leaving aside the issue of whether the frame affects dictator behaviour, our data suggest that the frame does have an important effect on how dictator decisions are judged by others.

We found that females made stronger attributions of harmful intent than males. This result contradicts previous studies using the Ambiguous Intentions Hostility Questionnaire^9^ where males report stronger hostile intention attributions among males than females^9, 56^, although it is possible that differences between rating notional written scenarios (questionnaire studies) and being actively engaged in a situation (the present study) may lead to the reported differences. Further research beyond the scope of the current study are necessary to explain this unexpected gender effect in more detail and we do not attempt to do so here.

Some limitations of this study also need to be borne in mind. Although participants in MTurk studies have been found to be more representative of the general population than typical lab studies with better attention to study participation^30^ they are still unrepresentative in some important ways – namely being younger, more highly educated and less likely to be in full time employment. Mechanical Turk workers are also more likely to be socially anxious though not more likely to report clinically relevant emotional dysregulation than the US population^57^. Moreover, we did not request information about history of contact with mental health services, drug or alcohol use, history of head injury, neurological problems, or trauma history. The extent to which our study represents paranoia as ‘unfounded’ fears about harm rather than a reaction to a history of harm or genuine threat is therefore difficult to determine, although we note that the high scorers on the GPTS paranoia scale scored within the clinical range^32^ However, the generalisability of these findings to people affected by paranoia in clinical services is not clear, as they may differ even from people with similar levels of paranoia in the general population without contact with clinical services in some important ways, most notably in terms of the use of medication. Consequently, further research using this approach needs to be conducted in collaboration with patients affected by paranoia and paranoid delusions to make the findings most relevant to clinically relevant experiences and situations.

## Conclusions

In summary, we present a large scale study involving a genuine social situation in which real resources were at stake, the results of which suggest that attributions of harmful intent are independent of immediate harm in paranoia. As such, we argue that the social representation of others plays an important but neglected role in paranoia and needs to be understood alongside the more traditional focus on immediate self-focused threat biases. The fact that this has attracted so little research interest is, perhaps, surprising, given that the spectrum of paranoia stretches from suspicion of genuine people to being bothered by delusional persecutors who are, by definition, illusory social agents^45^. We also note that we have found more paranoia-typical attributions using an experimental approach where highly paranoid people are engaged in genuine social interactions than in previous written vignette rating studies, suggesting paranoid thinking may be more readily studied in these more ecologically valid paradigms.

## Electronic supplementary material

Supplementary information
