# Supplementary material for: Paranoia and the social representation of others: a large-scale game theory approach
Source: Sci Rep. 2017 Jul 3;7:4544. doi: 10.1038/s41598-017-04805-3 (PMC5495777; doi:10.1038/s41598-017-04805-3)
Supplement: Supplementary file 1 — Supplementary information [file 41598_2017_4805_MOESM1_ESM.pdf]

Supplementary Information

Paranoia and the social representation of others: a large-scale game theory approach

Author names and affiliations:
Nichola J. Raihani[a], Vaughan Bell[b,c*]

a. Department of Experimental Psychology, University College London.
b. Division of Psychiatry, University College London.
c. South London and Maudsley NHS Foundation Trust

S1. The Green et al. (2008) Paranoid Thoughts Scale survey.
S2. The game instructions used in different versions of the Dictator Game.
S3. Supplementary Tables

S1. The Green et al. (2008) Paranoid Thoughts Scale

To begin, **please enter your Amazon Mechanical Turk WorkerID** here:
(Please see below for where you can find your Worker ID.)
Your Worker ID starts with the letter A and has 12-14 letters or numbers. It is NOT your email address. If we do not have your correct Worker ID we will not be able to pay you.

Your WorkerID can be found on your dashboard page.

You are about to take part in an academic study which is run by the Raihani Lab, based at University College London. This project has been approved by the UCL Ethics Board project 3720/001.
By continuing, you are consenting to allow the Raihani Lab to use your responses in the study for academic purposes.
The purpose of this study is to understand people's behaviour. All data are anonymous (your name will not appear in any publication related to this study and will not be shared with any other parties). By completing this HIT you will be granted a qualification to participate in a subsequent HIT run by Raihani Lab in the future. You will be notified when the next HIT becomes available.
Please tick 'I agree' if you agree to these conditions. If you do not wish to participate, or if you change your mind during the course of the study, please close this window.
❍ I agree (1)

Please read each of the following statements carefully. They refer to thoughts and feelings you may have had about others **over the last month**. Think about last month and indicate the extent of these feelings from **1 (Not at all) to 5 (Totally).**
Please complete both Part A and Part B.
(N.B. Please do not rate items according to any experiences you may have had under the influence of drugs.)

Part A:

	Not at all (1)	2	3	4	Totally (5)
1. I spent time thinking about friends gossiping about me.	❍	❍	❍	❍	❍
2. I often heard people referring to me.	❍	❍	❍	❍	❍
3. I have been upset by friends and colleagues judging me critically.	❍	❍	❍	❍	❍
4. People definitely laughed at me behind my back.	❍	❍	❍	❍	❍
5. I have been thinking a lot about people avoiding me.	❍	❍	❍	❍	❍
6. People have been dropping hints for me.	❍	❍	❍	❍	❍
7. I believed that certain people were not what they seemed.	❍	❍	❍	❍	❍
8. People talking about me behind my back upset me.	❍	❍	❍	❍	❍
9. I was convinced that people were singling me out.	❍	❍	❍	❍	❍
10. I was certain that people have followed me.	❍	❍	❍	❍	❍
11. Certain people were hostile towards me personally.	❍	❍	❍	❍	❍
12. People have been checking up on me.	❍	❍	❍	❍	❍
13. I was stressed out by people watching me.	❍	❍	❍	❍	❍
14. I was frustrated by people laughing at me.	❍	❍	❍	❍	❍
15. I was worried by people's undue interest in me.	❍	❍	❍	❍	❍
16. It was hard to stop thinking about people talking about me behind my back.	❍	❍	❍	❍	❍

Part B:

	Not at all (1)	2	3	4	Totally (5)
1. Certain individuals have had it in for me.	❍	❍	❍	❍	❍
2. I have definitely been persecuted.	❍	❍	❍	❍	❍
3. People have intended me harm.	❍	❍	❍	❍	❍
4. People wanted me to feel threatened, so they stared at me.	❍	❍	❍	❍	❍
5. I was certain people did things in order to annoy me.	❍	❍	❍	❍	❍
6. I was convinced there was a conspiracy against me.	❍	❍	❍	❍	❍
7. I was sure someone wanted to hurt me.	❍	❍	❍	❍	❍
8. I was distressed by people wanting to harm me in some way.	❍	❍	❍	❍	❍
9.I was preoccupied with thoughts of people trying to upset me deliberately.	❍	❍	❍	❍	❍
10. I couldn't stop thinking about people wanting to confuse me.	❍	❍	❍	❍	❍
11. I was distressed by being persecuted.	❍	❍	❍	❍	❍
12. I was annoyed because others wanted to deliberately upset me.	❍	❍	❍	❍	❍
13. The thought that people were persecuting me played on my mind.	❍	❍	❍	❍	❍
14. It was difficult to stop thinking about people wanting to make me feel bad.	❍	❍	❍	❍	❍
15. People have been hostile towards me on purpose.	❍	❍	❍	❍	❍
16. I was angry that someone wanted to hurt me.	❍	❍	❍	❍	❍

S2. Dictator Game Instructions

To begin, please enter your Amazon Mechanical Turk WorkerID here:
(Please see below for where you can find your Worker ID.)
Your Worker ID starts with the letter A and has 12-14 letters or numbers. It is NOT your email address. If we do not have your correct Worker ID we will not be able to pay you.

You are about to take part in an academic study which is run by the Raihani Lab, based at University College London. This project has been approved by the UCL Ethics Board project 3720/001.
Please note that Raihani Lab does not use deception. All participants are real.

By continuing, you are consenting to allow the Raihani Lab to use your responses in the study for academic purposes. The purpose of this study is to understand people's behaviour. All data are anonymous (your name will not appear in any publication related to this study and will not be shared with any other parties).You will take part in two tasks, Task A and Task B. Please tick 'I agree' if you agree to these conditions. If you do not wish to participate, or if you change your mind during the course of the study, please close this window.
❍ I agree (1)

Task A
Receiver role; giving frame

You are Player 2.
You have been allocated $0.00.

Player 1 is your partner
Player1 was allocated a bonus of $0.50.

Player 1 could choose one of the following options:

- keep $0.50 and send $0.00 to you
- keep $0.25 and send $0.25 to you

Player 1 has already made their decision.

Task A
Receiver role; taking frame

You are Player 2.
You have been allocated $0.50.

Player 1 is your partner
Player1 was allocated a bonus of $0.00.

Player 1 could choose one of the following options:

- take $0.50 of your bonus and leave $0.00 to you
- take $0.25 of your bonus and leave $0.25 to you

Player 1 has already made their decision.

Task A
Observer role; giving frame
You are an observer to an interaction that has taken place between two other workers, Player 1 and Player 2.

Player 1 was allocated a bonus of $0.50.
Player 2 was allocated a bonus of $0.00.

Player 1 could choose one of the following options:

- keep $0.50 and send $0.00 to Player 2
- keep $0.25 and send $0.25 to Player 2

Player 1 has already made their decision.

Task A
Observer role; taking frame

You are an observer to an interaction that has taken place between two other workers, Player 1 and Player 2.

Player 1 was allocated a bonus of $0.00.
Player 2 was allocated a bonus of $0.50.

Player 1 could choose one of the following options:

- take $0.50 of Player 2's bonus and leave $0.00 for Player 2
- take $0.25 of Player 2's bonus and leave $0.25 for Player 2

Player 1 has already made their decision.

Sample comprehension questions
First, we would like you to answer a few questions to ensure you have understood the game. Please note that you will only be allowed to proceed in this task and receive your bonus if you answer these questions correctly.

Q1. What was Player 1's starting bonus?
- ❍ $10 (1)
- ❍ $5 (2)
- ❍ $0.00 (3)

If $0.00 Is Not Selected, Then Skip To End of Block

Q2. If Player 1 took $0.25 from Player 2, how much would Player 2 keep?
- ❍ $0.25 (1)
- ❍ $0.50 (2)
- ❍ $0.00 (3)

If $0.25 Is Not Selected, Then Skip To End of Block

If dictator was fair, the following information was shown:
Player 1 decided to take $0.25 from Player 2 and leave $0.25 for Player 2.
That means Player 1 gets $0.25 and Player 2 also gets $0.25.

If dictator was unfair, the following information was shown:
Player 1 decided to take $0.50 from Player 2 and leave $0.00 for Player 2.
That means Player 1 gets $0.50 and Player 2 gets $0.00.

Please use the slider below to indicate the extent to which you believe Player 1's decision was driven by their **desire to earn money** in the game.

Please use the slider below to indicate to what extent Player 1's decision was driven by their desire **to reduce Player 2's bonus** in the game.

Task B
Now, you are taking part in the same sort of game.
You are Player 1. You have been allocated $0.50 bonus.
Your partner is Player 2. Player 2 has been allocated $0.00 bonus.

Your worker ID will not be revealed to the other player and you will not find out their worker ID.

You can choose to send none or half of your bonus to Player 2.
For example:

- if you choose to send $0.00, then you will keep $0.50 and Player 2 will get $0.00.
- if you choose to send $0.25, then you will keep $0.25 and Player 2 will get $0.25

Please indicate your decision below.
❍ I want to keep $0.50 and send $0.00 to Player 2 (1)
❍ I want to keep $0.25 and send $0.25 to Player 2 (2)

Thanks - it's nearly the end! Before you go, it would be very helpful if you could answer some additional questions to help with our research. Unlike some other research labs, Raihani Lab does not use deception on MTurk. All participants are real. Nevertheless, for our own purposes, it is helpful to know to what extent you believed that the other player really existed.

	1 - Very skeptical that other player was real (1)	2 (2)	3 (3)	4 (4)	5 -Very confident that other player was real (5)
Please choose one (1)	❍	❍	❍	❍	❍

Q36 To what extent have you participated in HITs similar to this before?

	1 - Nothing like this scenario (1)	2 (2)	3 (3)	4 (4)	5 - Exactly like this scenario (5)
Please choose one (1)	❍	❍	❍	❍	❍

Q37 What is your age?

Q38 What is your gender
❍ Male (1)
❍ Female (2)

Thanks for taking part - that's the end of the task. In Task A, you simply evaluated the decision made by another worker. In Task B, you took part in the role of Player 1 and made a decision about how much money you and Player 2 would get. In Task B, we also cast you in the role of Player 2 with a different worker in this game. Therefore your bonus will depend on your own decision in the Player 1 role - and your other partner's decision when you were in the Player 2 role. Once the HIT is completed we will compute bonuses and pay all workers. Bonuses will be paid within 10 days of the entire batch being completed. In order to receive your bonus, please be sure to enter the mystery word below in your HIT window before submitting your HIT.

Thanks for playing! If you would like to know more about the motivation and the outcome of this study, then please send an email to Raihanilab@gmail.com, with the subject line: "Tell me more".

Rank	Parameters	df	AICc	w^i
**1**	**Age + Fairness + Frame + Gender + Paranoia + Role + Fairness:Frame + Frame:Paranoia + Paranoia:Role**	**13**	**4952.4**	**0.21**
2	Age + Fairness + Frame + Gender + Paranoia + Fairness:Frame + Frame:Paranoia	11	4952.7	0.17
3	Age + Fairness + Frame + Gender + Paranoia + Role + Fairness:Frame + Fairness:Paranoia + Frame:Paranoia + Paranoia:Role	14	4953.3	0.13
4	Age + Fairness + Frame + Gender + Paranoia + Role + Fairness:Frame + Fairness:Role + Frame:Paranoia + Paranoia:Role	14	4953.4	0.13
5	Age + Fairness + Frame + Gender + Paranoia + Role + Frame:Paranoia + Paranoia:Role	12	4953.7	0.11
6	Age + Fairness + Frame + Gender + Paranoia + Fairness:Frame + Fairness:Paranoia + Frame:Paranoia	12	4954.1	0.09
7	Age + Fairness + Frame + Gender + Paranoia + Role + Fairness:Frame + Frame:Paranoia	12	4954.1	0.09
8	Age + Fairness + Frame + Gender + Paranoia + Frame:Paranoia	10	4954.3	0.08

Table S1. The top model set for the ordered logistic regression investigating the attribution of harmful intent to the dictator. Models are ranked in decreasing order of the support, based

on the relative AICc values. Akaike weights (w^i) give the probability that each model is the true best model.

Rank	Parameters	df	AICc	w^i
**1**	**Fairness + Frame + Paranoia + Fairness:Frame + Fairness:Paranoia**	**9**	**4643.5**	**0.37**
2	Fairness + Frame + Paranoia + Role + Fairness:Frame + Fairness:Paranoia	10	4645.0	0.18
3	Fairness + Frame + Paranoia + Fairness:Frame + Fairness:Paranoia + Frame:Paranoia	10	4645.0	0.17
4	Fairness + Frame + Gender + Paranoia + Fairness:Frame + Fairness:Paranoia	10	4645.4	0.14
5	Age + Fairness + Frame + Paranoia + Fairness:Frame + Fairness:Paranoia	10	4645.5	0.14

Table S2. The top model set for the ordered logistic regression investigating attributions of self-interest for dictators. Models are ranked in decreasing order of the support, based on the relative AICc values. Akaike weights (w^i) give the probability that each model is the true best model.
